# An Account of the Successful Extirpation of a Remarkable Schirrus of the Scrotum

**Published:** 1787

**Authors:** Richard Hall

**Affiliations:** Surgeon to the Manchester Infirmary


X. *An Account of the ſucceſsful Extirpation of a remarkable Schirrus of the Scrotum. Communicated in a Letter to Dr.* Simmons *by Mr.* Richard Hall, *Surgeon to the Mancheſter Infirmary.*

THOMAS Rhodes, fifty years of age, a very muſcular man, and of a healthy aſpect, was admitted into the Mancheſter Infirmary, on the 17th of October, 1785. He gave this account of himſelf: that between ten and eleven years ago, he had a ſmall indolent tumor formed in the coats of the ſcrotum, on the left ſide, totally independent of the teſticle, and which grew to the ſize of a hazel nut, when by means of ſome applications it rather leſſened, but ſoon began gradually to enlarge again, ſo that it now hung down below his knees.

The burden was become ſo inconvenient, and at times ſo painful, that he was very anxious for the removal of it. He walked about with ſeemingly much freedom, and could raiſe and move it to any direction. It was remarkable, that he could never wear a ſuſpenſory, as it occaſioned ſo much pain in the tumor.

He made little complaint of any uneaſineſs in the loins, but chiefly in the abdominal muſcles, and was then ſubject to colic pains. On the lower and poſterior part was a ſore, which diſcharged a very fœtid ichor.

The parts which ought to cover the os pubis were drawn down conſiderably below it; and the right abdominal ring was ſo dilated, as to occaſion a large hernia. The ſpermatic chords ſeemed to be free from diſeaſe; the penis was entirely buried; but he voided his urine freely.

The dimenſions of the tumor, the figure of which was rather irregular, were as follows, viz.

From the os pubis to where the præputium appeared, thirteen inches and a half:

From the os pubis to the lower extremity of the tumor, twenty-two inches and a half:

Leſſer circumference of the tumor, below the os pubis, eighteen inches:

Largeſt circumference, three feet four inches.

After extirpation, and when free from all fluid contents, it was found to weigh thirty-fix pounds and a half.

The patient lay upon a table, to which was fixed the back part of a wooden chair, which fupported the tumor. The operation proved tedious, from the neceffary flownefs, and fecuring the veffels as they were divided; notwithftanding which, he loft a good deal of blood; fo that he fainted frequently, and was once convulfed.

I began the incifion on the right fide, about fix inches below the os pubis, carrying it nearly to the center of the tumor, and from thence brought it down in a ftraight line to where the prepuce appeared, which led me to a difcovery of the right fpermatic chord and penis. The latter I carefully diffected out, leaving a fmall part of the prepuce to it. The incifion being then carried acrofs as at firft, expofed the left fpermatic chord, and I foon found both tefticles entirely free from difeafe: but as they were denuded, and future bad confequences were apprehended, it was thought prudent to remove them. This I did by making ligatures round the chords, and then dividing them with the fcalpel; and from this method I was pleafed to find no inconvenience enfue. I was then employed in diffecting away the fchirrus as clean as poffible from the fkin, of which I left fo much, as to completely cover the wound, and the edges were fecured by ligatures and fticking plafter: but the penis was fo much elongated, as to be left quite bare.

For three or four days after the operation he had a good deal of fever, which abated on the coming on of the digeftion, which was very good on the fifth day, and the union of the fkin began then to take place. The penis gradually contracted, and the prepuce ferved for a point of cicatrization, meeting with that from the parts above; fo that the whole was completely healed, and my patient was difcharged, cured, Dec. 26th, in nine weeks from the time of the operation.

He now, at the expiration of a year, remains perfectly well, except for the hernia, which is lefs, and for which he wears a trufs.

I believe this to be a very fingular cafe, and can no where find mention made of the extirpation of any thing fimilar to it. Many cafes are given us of enlarged fcrotums, or rather of farcoceles, in all of which the tefticles were difeafed, and gave origin to the complaint; whereas, whereas, here the difeafe was entirely confined to the fcrotum, which was a true fchirrous mafs, fo hard as to refift all impreffion of the hand. This tumor bore a great refemblance to that of the negro mentioned by Chefelden *; but as that was occafioned by the kick of a horfe, it is probable that one or both tefticles had received an injury: and the very remarkable one defcribed by Dr. Schotte †, was fuppofed to be an endemial difeafe of the tefticles.

*Manchefter,*

January 30, 1787.

---

XI. *An Account of a curious Fact relative to the Effects of crude Mercury. Communicated in a Letter to Dr.* Simmons *by* Michael Underwood, *M. D. Phyfician to the Britifh Lying-in Hofpital, and Licentiate in Midwifery of the Royal College of Phyficians, London.*

ALTHOUGH the internal exhibition of crude mercury has not been without its advocates, ever fince its introduction into me-

* Anatomy of the Human Body. Fourth edition. Tab. 26.

† See the Philof. Tranf. Vol. LXXIII. and the London Medical Journal, Vol. V.

